# The Frequency-Variable Rotor-Blade-Based Two-Degree-of-Freedom Actuation Principle for Linear and Rotary Motion

**DOI:** 10.3390/s23198314

**Published:** 2023-10-08

**Authors:** Xiaotao Li, Shengjiang Wang, Xiangyou Peng, Guan Xu, Jingshi Dong, Fengjun Tian, Qiuyu Zhang

**Affiliations:** 1 School of Mechanical and Aerospace Engineering, Nanling Campus, Jilin University, Changchun 130025, China; lixiaotao@jlu.edu.cn (X.L.); wangsj22@mails.jlu.edu.cn (S.W.); pengxy22@mails.jlu.edu.cn (X.P.); dongjs@jlu.edu.cn (J.D.); tianfj@jlu.edu.cn (F.T.); qyzhang22@mails.jlu.edu.cn (Q.Z.); 2 Transportation College, Nanling Campus, Jilin University, Changchun 130025, China

**Keywords:** piezoelectric actuation, frequency-variable regulation, two-degree-of-freedom, driving principle

## Abstract

Piezoelectric accurate actuation plays an important role in industrial applications. The intrinsic frequency of previous actuators is invariable. However, variable frequency can approach the range near the low-intrinsic-frequency and realize a high actuation capability. The frequency-variable linear and rotary motion (FVLRM) principle is proposed for rotor-blade-based two-degree-of-freedom driving. Inertial force is generated by frequency-variable piezoelectric oscillators (FVPO), the base frequency and vibration modes of which are adjustable by the changeable mass and position of the mass block. The variable-frequency principle of FVPO and the FVLRM are recognized and verified by the simulations and experiments, respectively. The experiments show that the FVLRM prototype moves the fastest when the mass block is placed at the farthest position and the prototype is at the second-order intrinsic frequencies of 42 Hz and 43 Hz, achieving a linear motion of 3.52 mm/s and a rotary motion of 286.9 mrad/s. The actuator adopts a lower operating frequency of less than 60 Hz and has the function of adjusting the natural frequency. It can achieve linear and rotational motion with a larger working stroke with 140 mm linear movement and 360° rotation.

## 1. Introduction

In recent years, research on micro-electro-mechanical systems (MEMS) has developed. As an important part of MEMS research, accurate actuation has become a hotspot in this field. Piezoelectric ceramics have the advantages of a fast response, high displacement resolution, high structural stiffness, and miniaturization [1,2], so piezoelectric ceramic actuators have broad application prospects in the field of precision drive [3,4]. They have a wide range of applications in many aspects, such as biomedicine [5,6,7], ultra-precision machining [8,9], precision optics [10,11], micro- and nano-positioners [12,13], triboelectricity [14], and microscopes [15,16,17], etc. The current mainstream research on piezoelectric precision drives focuses on stepper-type piezoelectric actuation [18,19,20], ultrasonic-type piezoelectric actuation [21,22], and inertial impact piezoelectric actuation [23,24]. Inertial impact piezoelectric actuation has received much attention, due to the distinctive actuation method of the inertial force changing the drive force and positive pressure for fast motion, a high resolution, a large stroke, and a simple structure [25,26].

In 2018, Yokozawa, Hiroki et al. [27] investigated a resonant smooth impact drive mechanism (R-SIDM) actuator fabricated from lead-free piezoelectric materials. The R-SIDM actuator is a linear ultrasonic motor driven by a sawtooth wave signal. In 2021, Yang et al. [28] proposed a new compact piezoelectric stick-slip actuator based on the ultrasonic friction reduction effect. The actuator provides a large deformation for stick-slip motion using a piezoelectric stack driven by asymmetric sawtooth wave, which can achieve only one-degree-of-freedom (1D). In 2020, Huang et al. [29] presented a stick-slip piezoelectric actuator with an L-shaped flexible hinge to achieve 1D motion. The actuator can achieve higher motion speeds with sawtooth wave excitation at an operating frequency of 800 Hz. In 2023, Sun et al. [30] explored an inertial impact piezoelectric actuator with a sawtooth wave signal. The actuator is driven by an asymmetric flexure mechanism with one thick end and one thin end to generate asymmetric friction between the drive foot and the U-slot, which also allows for 1D motion. In 2021, Pinskier, Joshua et al. [31] contributed a fully stick-slip positioner with error compensation. It employs sawtooth wave actuation and can be used for remote, precision-compatible mechanisms. In 2021, Xu et al. [32] proposed a bionic inertial piezoelectric actuator with the sawtooth wave. The experimental results showed that it achieved 1D motion with maximum horizontal and vertical loads of 0.6 N and 17 N, respectively. In 2021, Hu et al. [33] outlined a new linear motor with two motion modes on the basis of the piezoelectric inertial impact principle, which is also driven by the sawtooth wave. In 2019, Zhang et al. [34] proposed a piezoelectric motor that can achieve rotary motion around two orthogonal axes. The measurement results showed that the maximum rotor speed was 0.153 rad/s around the *x*-axis and 0.154 rad/s around the *y*-axis at a 400 V voltage and 460 Hz frequency, respectively. In 2020, a stick-slip piezoelectric rotary actuator was designed by Wang et al. [35]. The results showed that the maximum angular velocity of the actuator was about 55,000 μrad/s at the drive voltage of 100 V and the frequency of 600 Hz. In 2022, Li et al. [36] developed an inertial impact motion robot that can perform planar motion with three degrees of freedom (DOFs), including translation along the *x*-axis, translation along the *y*-axis, and rotation around the *z*-axis. In 2022, Liu et al. [37] proposed a bonded, longitudinal bending hybrid 2 DOF ultrasonic rotary actuator, which can achieve a maximum angular velocity of 414 deg/s in the *x* and *y* directions under an excitation voltage of 550 Vpp. In 2023, Huang et al. [38] developed a new type of 2 DOF micro-positioning platform that can realize linear and rotational motion, with a workspace of 22.90 mrad and 95.03 μm.

Previous studies of actuators have mainly realized unidirectional motion, and there are a few actuators that can move both linearly and rotationally. Most actuators have become complex and less feasible due to the increment in degrees of freedom. Thus, piezoelectric actuators with a relatively simple structure for multiple degrees of freedom have been a hot research topic in the field. The piezoelectric element generates heat at the resonant frequency due to the low mechanical factor, which reduces the reliability of the actuation, so inertial impact piezoelectric actuation generally vibrates at quasi-resonant conditions. Hence, previous actuators have mainly worked near the fixed and high natural frequency of the piezoelectric system. Although the performance of the actuator can be maximized near the high resonant frequency, it is easy to damage it due to piezoelectric wafer fatigue, and its lifetime is generally shortened. Moreover, the high resonant frequency of the actuator enlarges the vibration noise in the motion. Additionally, previous actuators with the fixed natural frequency of inertial impact piezoelectric actuation cannot provide the appropriate frequency for the situation with the connected load on the piezoelectric system, which changes the natural frequency of the original piezoelectric system. For example, in the angle adjustment for 3D laser cutting, and in the translation or rotation of the stage of the microscope, the piezoelectric actuator and the external load form a vibration system. The natural frequency of the vibration system is different from the one of the piezoelectric actuator. At this time, it is necessary to adjust the natural frequency of the piezoelectric actuator system to adapt to the driving electrical signal. Furthermore, for the fixed-frequency excitation signal, it is necessary to achieve the quasi-resonant condition for large displacement and rapid movement by adjusting the quasi-resonant frequency of the actuator to the excitation signal frequency. Therefore, it is a challenge and also important to flexibly realize variable- and low-frequency regulation under the corresponding conditions for piezoelectric actuators.

The frequency-variable linear and rotary motion (FVLRM) principle is proposed for rotor-blade-based accurate two-degree-of-freedom actuation, which mainly adopts the piezoelectric element to work near the resonant frequency. On the one hand, the variable resonant frequency of the piezoelectric actuator is adjusted at the structural level. On the other hand, the resonant frequency of the inertial impact piezoelectric actuator works at the low frequency, which reduces the vibration noise to improve the working reliability. The main contributions and works are concluded as follows:

(1) The rotor-blade-based FVLRM is proposed, in order to study the variable- and low-frequency regulation for linear and rotary actuation.

(2) The influence and variation law of the mass block position and weight on the single-row-hole frequency-variable substrate (SRHFVS) are investigated. Simulations and experimental verifications are performed to explore the variation law and performance of frequency-variable piezoelectric oscillators (FVPO).

(3) The resonant frequency of the SRHFVS is adjusted and analyzed for the FVLRM actuation principle. The speed and displacement of linear and rotary motion are experimentally tested for the variable-frequency regulation and the actuation performance.

## 2. Simulation Analysis of SRHFVS-FVPO and FVLRM

### 2.1. FVLRM Structure

**Figure 1:**
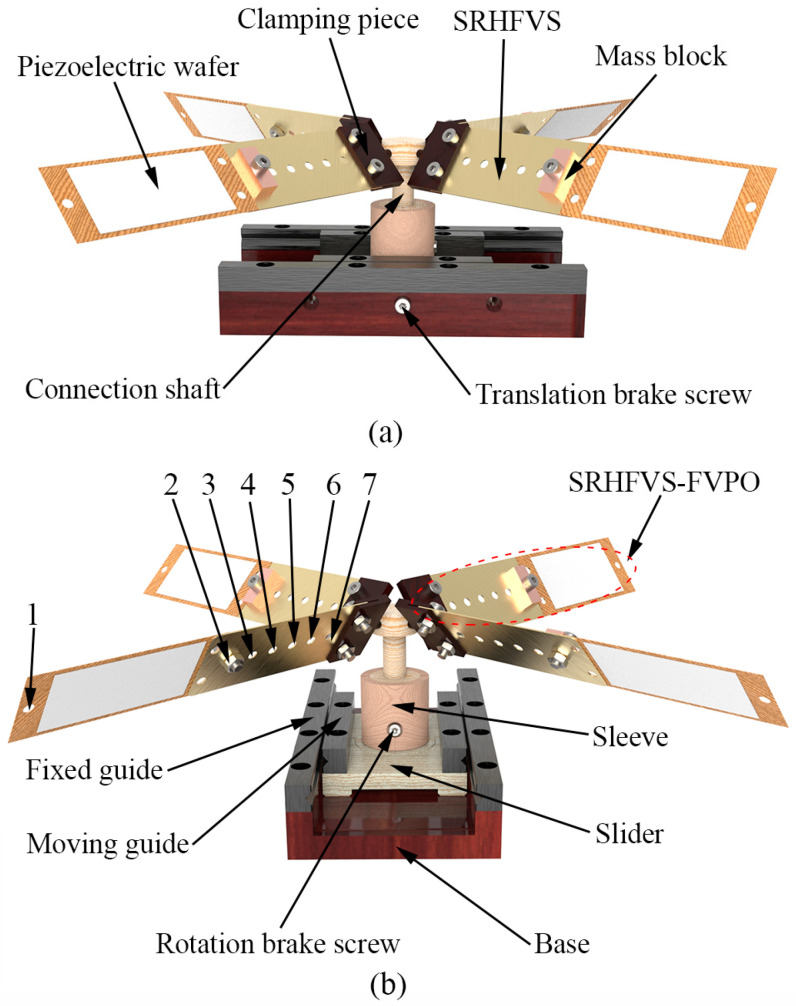
Structure of the FVLRM. 1–7 show the hole-positions. (**a**) Side view. (**b**) Front view.

The real actuation system of the FVLRM is shown in Figure 1, which includes a sleeve, a slider, a base, a fixed guide, a moving guide, a rotation brake screw, four SRHFVS-FVPOs, a connection shaft, and a translation brake screw. 

The angle of the SRHFVS-FVPO can be adjusted by the clamping piece. Two brake screws are adopted in the FVLRM model. One screw is for the linear motion. The other is for the rotation. The slider can move along the base and will be fixed to the base by the brake screw of linear motion. For the rotation, the rotation shaft is locked or unlocked by the rotation brake screw. Therefore, linear motion is achieved by the locked rotation brake screw and the unlocked brake screw of the linear motion. The rotation is achieved by the unlocked rotation brake screw and the locked brake screw of the linear motion. Four FVPOs are evenly arranged and fixed on the connecting shaft. The angle between the FVPO and horizontal plane is 45° or −45°.

As the actuation performance of the SRHFVS-FVPO is influenced by the resonant frequency, the SRHFVS-FVPO is designed to investigate the characteristics of the intrinsic frequency and vibration pattern of the FVPO with the SRHFVS, as well as to realize the frequency adjustment of the FVPO. As shown in Figure 1a, the SRHFVS-FVPO mainly consists of the clamping piece, the SRHFVS, the piezoelectric wafer, and the mass block. The dimensions of the FVPO are 144 mm × 33 mm × 7.25 mm. A row of seven circular holes is manufactured on the SRHFVS, with the positions 1–7 arranged as shown in Figure 1b, and fixed on the free end of the FVPO. The performance adjustment of the FVPO is realized by the variable distance of the mass block relative to the fixed end, i.e., to change the mass distribution condition of the substrate.

### 2.2. Simulation of SRHFVS-FVPO

Firstly, the simulation model of the single piezoelectric wafer and single-row-hole substrate is established in Figure 1. All parts of the single-substrate frequency-variable piezoelectric wafer are connected and then the material properties are defined. The material of the screws and nuts used is structural steel. As brass is a metal material easily manufactured with a good plasticity, strength, and toughness, the substrate, mass block, and SRHFVS are made of brass with a density of 8.27 × 10^3^ kg/m^3^, an elastic modulus of 99.95 GPa, and a Poisson’s ratio of 0.34. Piezoelectric material is an anisotropic physical material with a density of 7.95 × 10^3^ kg/m^3^, Poisson’s ratio of 0.34, and Young’s modulus of 66 GPa. The grid size of the SRHFVS-FVPO is set to 0.2 mm, and the other components of the FVLRM are set to 1 mm.

**Figure 2:**
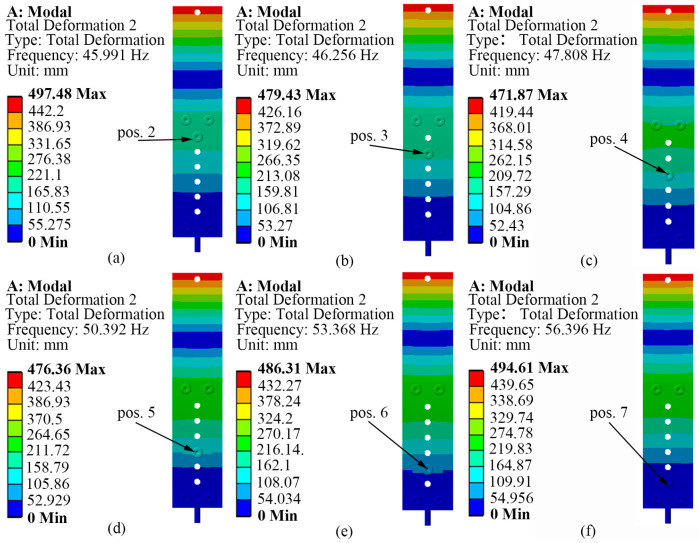
Second-order intrinsic frequency and vibration pattern of SRHFVS-FVPO with different mass block positions. (**a**–**f**) Response of SRHFVS-FVPO with the 6.6 g mass block at positions 2–7.

Since the actuator relies on the inertial drive, the first-order inherent frequency of the SRHFVS-FVPO is around 13 Hz according to the frequency sweep simulation, which provides a slow vibration speed for the insufficient inertial driving force. Therefore, the actuation frequency is selected near the second-order inherent frequency. In Figure 2, the circular handle of the clamping block is fixed by the face fixed constraint. It can be seen that a 6.6 g mass block is placed on the SRHFVS-FVPO, and its position is sequentially changed from position 2 to position 7. The corresponding second-order natural frequencies of the SRHFVS-FVPO are 45.99 Hz, 46.26 Hz, 47.81 Hz, 50.39 Hz, 53.37 Hz, and 56.40 Hz, respectively. It can be seen that the SRHFVS-FVPO has a relatively ideal mode at the second-order natural frequency, with the maximum deformation occurring at the free end and being able to obtain a large driving inertia force. As the mass block gets closer to the center of the fixed end, the second-order intrinsic frequency shows an obvious increment. Furthermore, the different weight of the block is also considered in the simulation model, in order to analyze the influence of the block weight on the intrinsic frequency. The mass block is placed at position 2 and set to 4.8 g, 6.6 g, and 8.4 g, respectively. The frequencies of the SRHFVS-FVPO are 47.12 Hz, 45.99 Hz, and 45.04 Hz. It can be seen that, as the weight of the mass block increases, the intrinsic frequency decreases. In order to further explain the simulation results, the FVPO is considered as a cantilever beam with an equivalent concentrate load *W* acting on the free end. The free end deformation *δ*_s_ and equivalent stiffness *k* are [39]

*δ*_s_ = (*Wl*^3^)/(3*EI*) (1)

*k* = *W*/*δ*_s_ = 3*EI*/*l*^3^ (2)

where *E* is the elastic modulus, *I* is the moment of inertia, and *l* is the distance from the equivalent concentrate load to the center of the fixed end. According to Equations (1) and (2), as the mass block gradually approaches the fixed end, *l* becomes smaller and *k* becomes larger. As the natural frequency is bigger when the stiffness is greater, the closer the mass block is to the fixed end, the higher the natural frequency of the FVPO.

### 2.3. Simulation Analysis of FVLRM

**Figure 3:**
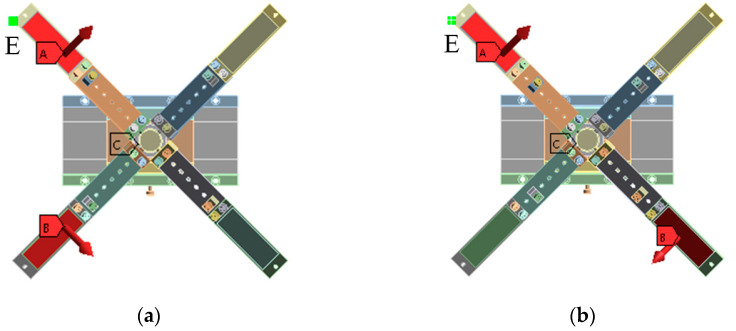
External excitation analysis of FVLRM with four SRHFVS-FVPOs. (**a**) Linear motion. (**b**) Rotary motion.

As the above SRHFVS-FVPO can be considered as the actuation source, the FVLRM designed in Figure 3 consists of four SRHFVS-FVPOs to achieve the rotation and translation. Thus, after the modal analysis of the SRHFVS-FVPO, a frequency sweep analysis of the actuation model of the FVLRM is required to determine its reasonable actuation frequency. The FVLRM moves along a straight line, which is realized by two neighbor SRHFVS-FVPOs, as shown in Figure 3a. The base, slider, sleeve, and connection shaft are made of hard aluminum with a density of 2.7 × 10^3^ kg/m^3^, an elastic modulus of 69 GPa, and a Poisson’s ratio of 0.3. Binding contact is used among various parts. An external excitation of 0.1 N is added to the two adjacent SRHFVS-FVPOs. The base is fixed by the face fixed constraint. The combined force on the horizontal plane follows the direction of the guide rail. The sweep frequency range is set to 0~70 Hz, and the frequency response is analyzed using the vertex E at the blade end. The rotary state of the FVLRM, as shown in Figure 3b, is generated from the external excitations of the opposite two SRHFVS-FVPOs. At this time, the combined force on the horizontal plane can provide the torque around the *z*-axis to rotate the rotor.

**Figure 4:**
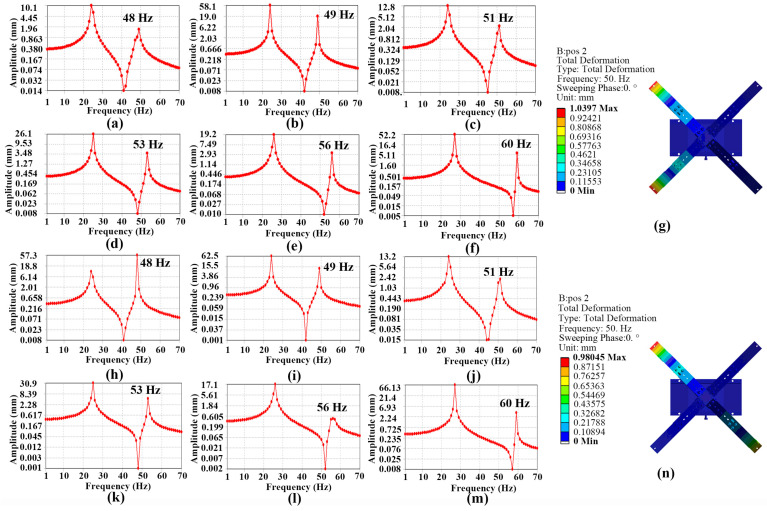
Frequency-variable simulation of the FVLRM. (**a**–**f**) Responses of the FVLRM for position 2–position 7 in linear motion. (**g**) Deformation of the FVLRM for the 6.6 g mass block at position 2 and the excitation frequency of 50 Hz in linear motion. (**h**–**m**) Responses of the FVLRM for position 2–position 7 in rotational motion. (**n**) Second−order intrinsic frequency of FVLRM for the 6.6 g mass block at position 2 in rotational motion.

In order to analyze the intrinsic frequency of the FVLRM about the different block mass and different positions, the simulation results of the FVLRM model are shown in Figure 4. The base of the FVLRM is fixed by the face fixed constraint. In Figure 4a–f, for the linear motion of the FVLRM, the second-order intrinsic frequencies of the FVLRM are 48 Hz, 49 Hz, 51 Hz, 53 Hz, 56 Hz, and 60 Hz, corresponding to the positions from 2 to 7 of the mass block. As the mass block moves towards the center of the rotation axis, the second-order natural frequency significantly increases, with a frequency modulation range of 48~60 Hz. Thus, it is clear that the resonant frequency of the FVLRM can be varied by different positions of the mass block. The vibration deformation of the FVLRM is shown in Figure 4g. The two working neighbor SRHFVS-FVPOs generate deformations and actuation forces. The sum of the actuation forces enables the FVLRM move along a determined direction. The left two neighbor SRHFVS-FVPOs do not provide deformations and actuation forces for the FVLRM. It can be seen that the FVLRM linear movement has a relatively ideal mode under the second-order natural frequency, with the maximum deformation occurring at the free end of two piezoelectric wafers. The simulation results of the FVLRM are similar to the analyses of the actuation element, SRHFVS-FVPO. Then, the mass block is placed at position 2 and set to 4.8 g, 6.6 g, and 8.4 g, respectively. The corresponding intrinsic frequencies of the FVLRM are 49 Hz, 48 Hz, and 47 Hz. It can be seen that, as the weight of the mass block increases, the intrinsic frequency decreases.

The rotation simulation of the FVLRM is also performed and the results are shown in Figure 4h–m. The base of the FVLRM is fixed by the face fixed constraint. The second-order resonant frequencies of the FVLRM in rotary motion are 48 Hz, 49 Hz, 51 Hz, 53 Hz, 56 Hz, and 60 Hz, which correspond to the positions of the mass block in the rotation. As the mass block approaches the center of the rotation axis, the second-order natural frequency increases, with a frequency modulation range of 48~60 Hz. The second-order intrinsic frequency grows as the mass block approaches the center of the rotation axis. The vibration pattern of the FVLRM is shown in Figure 4n. The two working opposite SRHFVS-FVPOs produce deformations and actuation forces, which enable the FVLRM to rotate around an axis. The second-order intrinsic frequency grows as the mass block approaches the center of the rotation axis. It can be seen that the FVLRM rotation has a relatively ideal mode under the second-order natural frequency. The intrinsic frequency and variation characteristics of the FVLRM in the rotary motion are similar to those of the linear motion. Then, the mass block is placed at position 2 and set to 4.8 g, 6.6 g, and 8.4 g, respectively. The corresponding intrinsic frequencies of the FVLRM are 49 Hz, 48 Hz, and 47 Hz. It can be seen that, as the weight of the mass block increases, the intrinsic frequency decreases.

## 3. Motion Analysis of FVLRM

### 3.1. FVLRM Rotation Sequence Analysis

**Figure 5:**
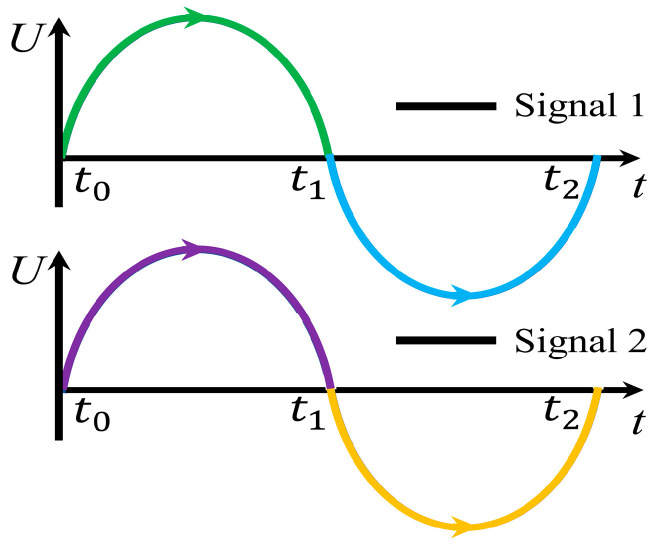
Excitation signal on two SRHFVS-FVPOs of FVLRM.

As the sinusoidal excitation signal changes smoothly and with a lower change rate, it is easy to be implemented for the actuation and also a benefit for the working life of the piezoelectric material. Thus, the sinusoidal excitation signal is used in the working principle analysis of the FVLRM. Figure 5 shows the waveform applied on the two SRHFVS-FVPOs of the FVLRM. The clockwise rotation of the FVLRM is achieved by the FVPO 1 and FVPO 4. The clockwise rotation can be divided into three steps.

Step 1: At the moment *t*_0_, the actuator remains stationary due to the zero voltage.

**Figure 6:**
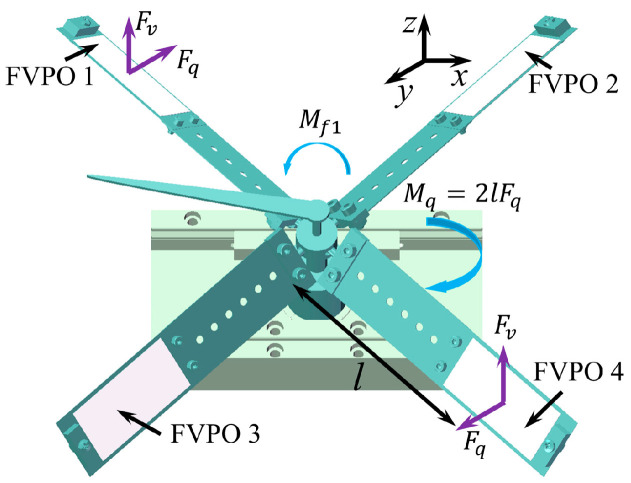
Force analysis for the rotation of FVLRM.

**Figure 7:**
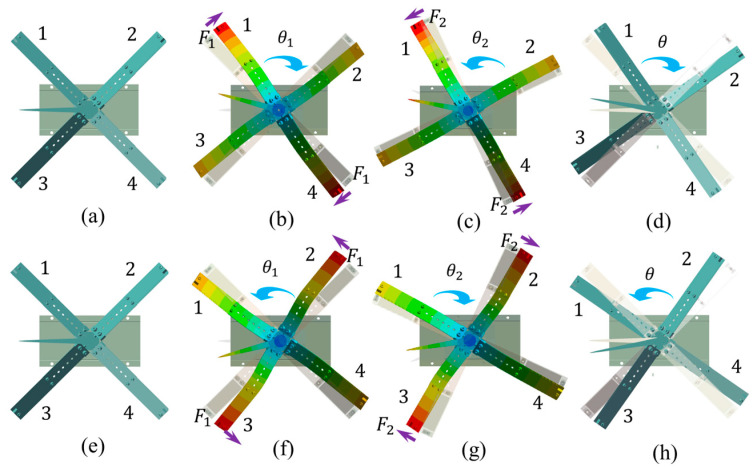
Motion analysis for the rotation of FVLRM. 1–4 are four blades. (**a**) *θ* = 0°. (**b**) *θ* = *θ*_1_. (**c**) *θ* = *θ*_2_. (**d**) *θ* = *θ*_1_ − *θ*_2_. (**e**–**h**) Counterclockwise rotation of (**a**–**d**).

Step 2: During *t*_0_–*t*_1_, FVPO 1 and FVPO 4 generate the inertial force perpendicular to the wafer after the voltage is added, as shown in Figure 6. The approximate deformation of the FVLRM is shown in Figure 7b. The force can be decomposed into horizontal and vertical components. The two horizontal components provide a clockwise driving moment *M_q_*. The two vertical components and gravity make the counterclockwise friction resistance moment *M_f_*_1_ between the contact surfaces smaller, due to *M_q_* > *M_f_*_1_. The actuator therefore rotates one big step clockwise, noted by *θ*_1_.

Step 3: During *t*_1_–*t*_2_, since the drive signal changes from positive to negative at this time, the direction of the combined driving moment *M_q_* also becomes counterclockwise, and the frictional resistance moment between the contact surfaces in the clockwise direction becomes a larger *M_f_*_2_. The deformation of the FVLRM at this time is shown in Figure 7c. As *M_q_* > *M_f_*_2_ and *M_f_*_2_ > *M_f_*_1_, the FVLRM rotates counterclockwise by one small step, which is recorded by *θ*_2_. The total displacement of the clockwise rotation in one cycle is *θ* = *θ*_1_ − *θ*_2_, as shown in Figure 7d.

The counterclockwise rotation relies on FVPO 2 and FVPO 3. The clockwise rotation and counterclockwise rotation of the FVLRM are achieved by the combination of the FVPOs, but the principles are exactly the same. The counterclockwise rotation is shown in Figure 7e–h.

### 3.2. FVLRM Translation Sequence Analysis

The forward linear motion of the FVLRM relies on FVPO 1 and FVPO 3. The waveforms used for the rotary and linear motion of the actuation are the same and are shown in Figure 5. The forward linear motion is divided into three steps.

Step 1: At the moment *t*_0_, the FVLRM is stationary because no voltage is applied.

**Figure 8:**
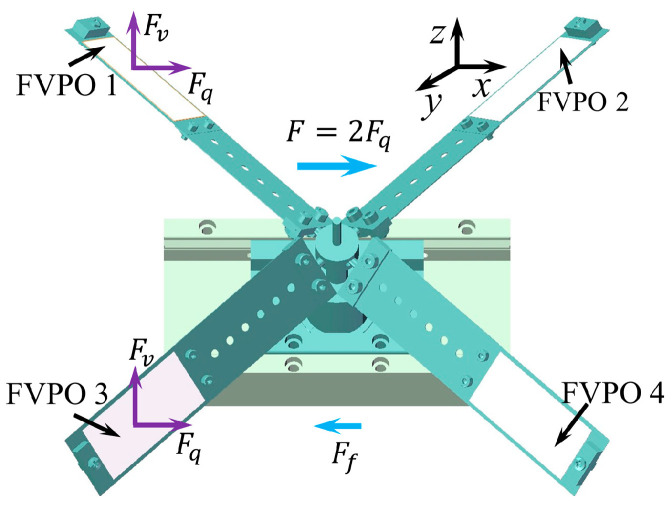
Force analysis for the translation of FVLRM.

**Figure 9:**
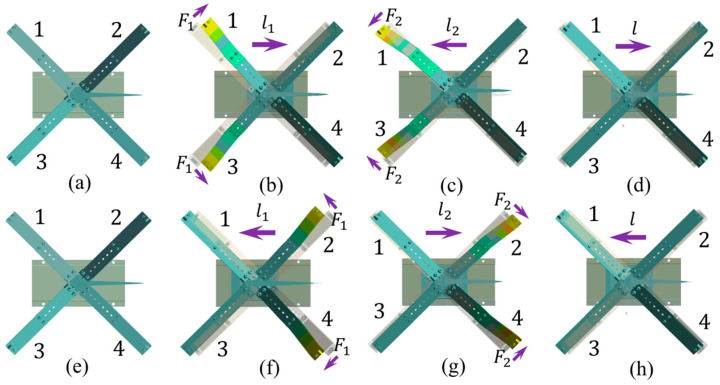
Motion analysis for the translation of FVLRM. 1–4 are four blades. (**a**) *l* = 0. (**b**) *l* = *l*_1_. (**c**) *l* = *l*_2_. (**d**) *l* = *l*_1_ − *l*_2_. (**e**–**h**) Backward translation of (**a**–**d**).

Step 2: During *t*_0_–*t*_1_, FVPO 1 and FVPO 3 generate the inertial force perpendicular to the wafer after adding the voltage, as shown in Figure 8. The inertial force can be decomposed into horizontal and vertical components. The two horizontal components will form a combined driving force *F* along the *x*-axis. The two vertical components make the frictional resistance *F_f_*_1_ between the contact surfaces along the *x*-axis negative direction smaller. The deformation of the FVLRM is shown in Figure 9b. Due to *F* > *F_f_*_1_, the FVLRM generates a big step forward along the *x*-axis, noted by *l*_1_.

Step 3: During *t*_1_–*t*_2_, as the input signal changes from positive to negative, and the direction of the driving force *F_q_* becomes negative along the *x*-axis. The frictional resistance between the contact surfaces along the positive *x*-axis becomes a bigger force *F_f_*_2_, and the deformation of the FVLRM is shown in Figure 9c. Meanwhile, as *F* > *F_f_*_2_ and *F_f_*_2_ > *F_f_*_1_, the FVLRM will take a small step backwards along the negative *x*-axis, which is recorded by *l*_2_. The total displacement of the actuation in one cycle to the positive direction of the *x*-axis is *l* = *l*_1_ − *l*_2_, as shown in Figure 9d.

The negative linear motion is realized by the FVPO 2 and FVPO 4. The negative linear motion is shown in Figure 9e–h. The positive and negative linear motion of the FVLRM is also realized by the combination between the FVPOs.

## 4. Frequency Regulation Test of FVPO and Experiment Analysis of FVLRM

### 4.1. Frequency Regulation Test of FVPO

In the sequence analysis of the FVLRM, the SRHFVS-FVPOs are the actuation source of the FVLRM. Thus, the vibration performance of the SRHFVS-FVPO should be studied for the actuation principle of the linear and rotary motion. According to the simulation analysis of the SRHFVS-FVPO, the displacement is experimentally analyzed with the same amplitude of driving voltage and different input signal frequencies. On this basis, a further experimental analysis of the variation law of the intrinsic frequency of the SRHFVS-FVPO is explored.

**Figure 10:**
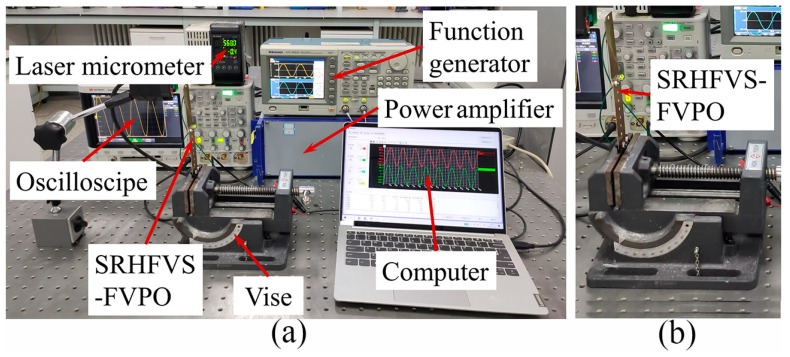
Test instrumentations of the SRHFVS-FVPO. (**a**) Measurement system configuration. (**b**) Clamping position of the SRHFVS-FVPO.

The test system for the frequency regulation experiment in Figure 10 includes a function generator, a power amplifier, an oscilloscope, a laser micrometer, a vise, a SRHFVS-FVPO, and a computer for data acquisition. The clamping piece of the FVPO is first fixed on the vise. Then, the function generator provides the excitation signal to the power amplifier, which enlarges the amplitude of the driving voltage for the FVPO. The frequency of the excitation signal can be adjusted by the function generator. The feedback electronic signal put on the FVPO is indicated by the oscilloscope. Finally, the displacement of the FVPO is measured by the laser micrometer and displayed on the computer.

**Figure 11:**
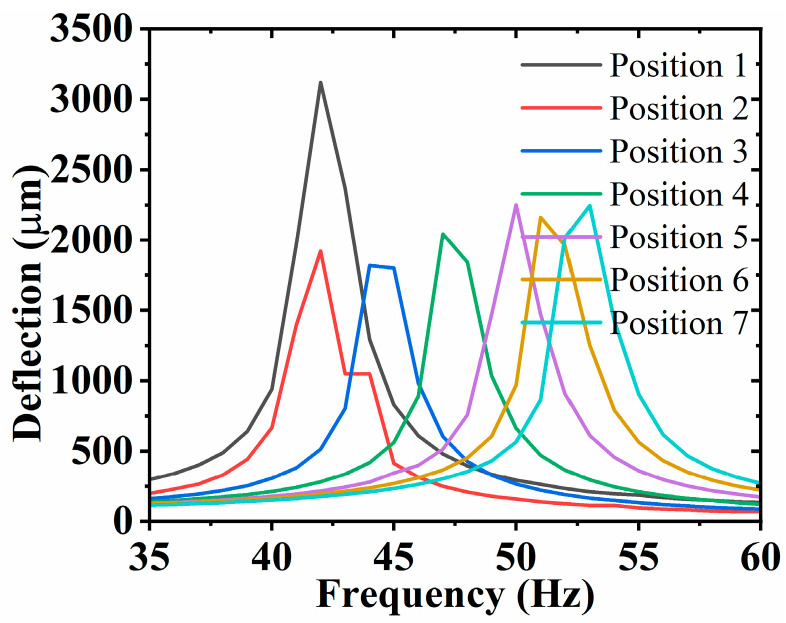
Deflection-frequency measurement results of SRHFVS-FVPO with the mass block at different positions.

The deflection at the end of the SRHFVS-FVPO is measured at the voltage of 88 V. The position of the mass block of the SRHFVS-FVPO is changed from position 1 to position 7, respectively. The input signal frequency ranges from 1 Hz to 60 Hz, and the experimental deflection results are shown in Figure 11. The second-order intrinsic frequency of the SRHFVS-FVPO decreases from 53 Hz to 41 Hz, with the increment in the distance between the mass block and the fixed end. The experimental results show that the intrinsic frequency of the SRHFVS-FVPO can be adjusted by the position of the mass block. And the second-order intrinsic frequency increases as the mass block gets closer to the clamping piece. The variation trend of the experimental results is similar to that of the simulation.

### 4.2. Experimental Instrumentations of FVLRM

**Figure 12:**
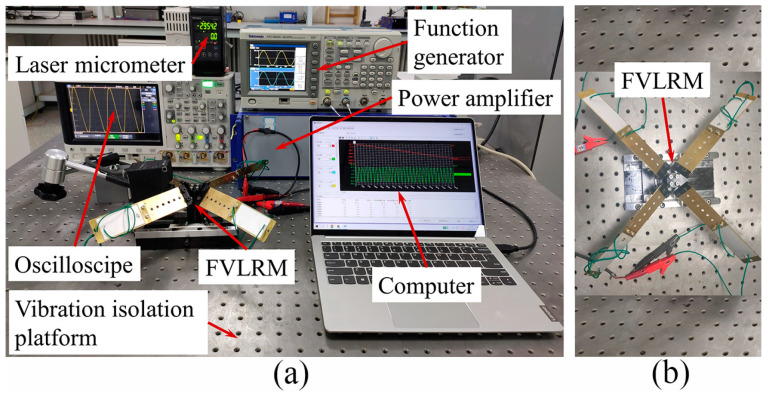
Test instrumentations of the FVLRM. (**a**) Measurement system configuration. (**b**) Fixation method of the FVLRM.

In order to further verify the rotation and linear motion characteristics of the FVLRM, the test system is constructed, as shown in Figure 12. The instrumentations of the test system include a function generator, a power amplifier, an oscilloscope, a laser micrometer, a vise, an FVLRM prototype, a vibration isolation platform, and a computer. The base of the FVLRM is fixed on the vibration isolation platform, which isolates the external vibration from the environment using the air-suspending system. In the experiment, the rotation and linear motion of the FVLRM should be independent of each other for a high actuation accuracy. Therefore, in the translation test, the linear motion is achieved by locking the rotation brake screw and unlocking the linear motion brake screw. In the rotation test, the rotation is generated by the unlocked rotation brake screw and the locked linear motion brake screw.

### 4.3. Rotation Characteristics of FVLRM

**Figure 13:**
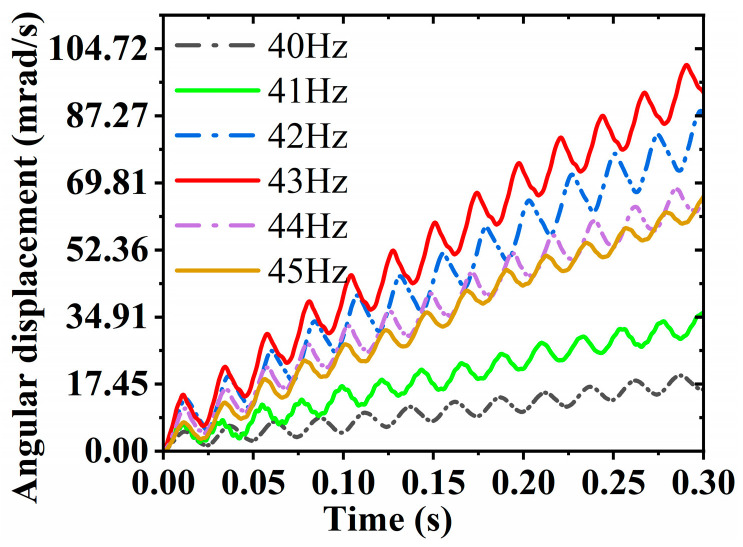
Angular displacement of the FVLRM rotation at different frequencies.

In the rotation characteristics study of the FVLRM, the frequency and voltage of the input signal are considered in the test. In the experiments, the FVLRM is capable of 360° rotation under the sinusoidal wave excitation. The angular displacement of the FVLRM about the time is tested using the sinusoidal wave excitation at the frequency of 40–45 Hz with a 1 Hz interval. The mass block is placed at position 1 of the FVPO with the SRHFVS, as shown in Figure 13. The average step of the FVLRM is the largest at the operating frequency of 43 Hz and the motion speed of the FVLRM is the fastest about different frequencies. However, it produces a large reverse regression, which decreases the positioning stability. In the operating frequency range around 43 Hz, there is a significant decrease in the motion speed. However, the positioning stability of the FVLRM is improved. The angular displacement rises slowly at operating frequencies below 42 Hz and the positioning stability is better. If a high positioning stability is required, the FVLRM should work in the operating frequency range below 42 Hz.

**Figure 14:**
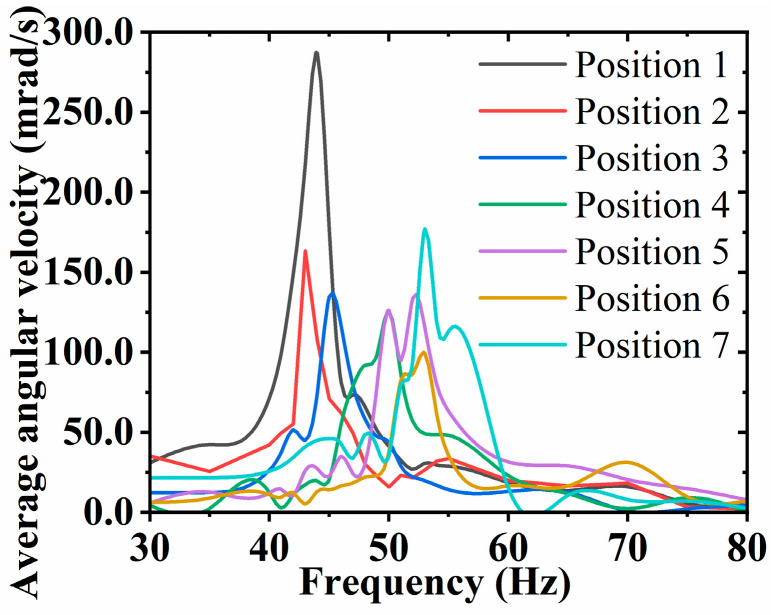
Average angular velocity of the FVLRM rotation with the mass block at different positions.

The average angular velocity of the FVLRM is tested by changing the position of the mass block and gradually increasing the frequency from 30 Hz to 80 Hz under the sinusoidal wave signal with a voltage of 88 V. The relationship between the angular velocity and frequency of the FVLRM rotation is shown in Figure 14. The mass block at position 1 provides the maximum rotational moment of inertia, which shows the maximum average angular velocity of 286.9 mrad/s. The intrinsic frequencies of the FVLRM are 43 Hz, 45 Hz, 50 Hz, 52 Hz, 53 Hz, and 54 Hz when the mass block is moved from position 2 to position 7. Hence, the position of the mass block can obviously change the intrinsic frequency of the FVLRM. The closer the mass block is to the center of the rotation axis, the larger the intrinsic frequency of the FVLRM is. The experimental results are similar to the simulation results.

### 4.4. Linear Displacement Characteristics of FVLRM

**Figure 15:**
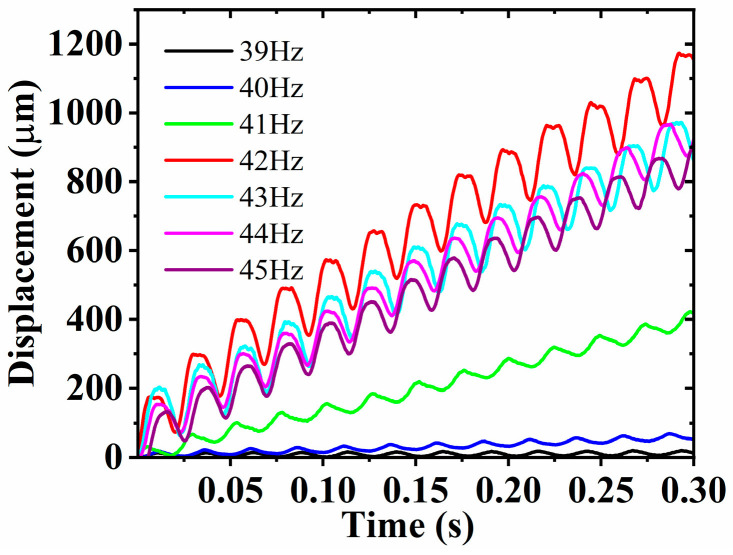
Linear displacement of the FVLRM at different frequencies.

The FVLRM is capable of forward and backward linear motion under the actuation of the input sinusoidal excitation. The displacement time curves of the FVLRM translation are tested by placing the mass block at position 1 of the SRHFVS-FVPO, as shown in Figure 15. The frequencies of the input sinusoidal wave excitation are 39 Hz, 40 Hz, 41 Hz, 42 Hz, 43 Hz, 44 Hz, and 45 Hz, respectively. From the effect of the different frequencies on the displacement curve of the FVLRM in Figure 15, the slope of the displacement curve gradually increases from 39 Hz to 41 Hz, and then decreases from 43 Hz to 45 Hz. The maximum slope of the displacement curve reaches its peak at 42 Hz.

**Figure 16:**
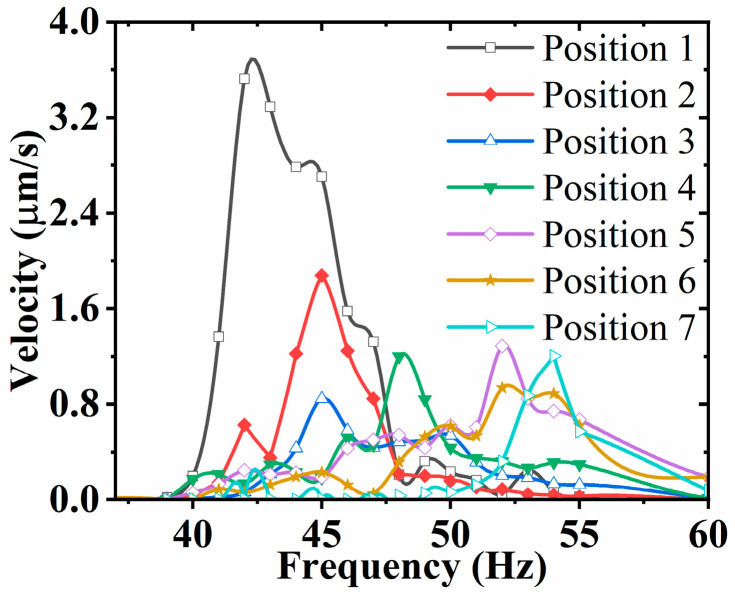
Average velocity of the FVLRM translation with the mass block at different positions.

For the input sinusoidal signal, the frequency gradually increases from 35 Hz to 60 Hz. The position of the mass block on the SRHFVS-FVPO is changed and the average velocity of the FVLRM varies with the frequency, as shown in Figure 16. When the mass block is moved from position 2 to position 7, the position of the mass block can obviously change the intrinsic frequency of the FVLRM. The intrinsic frequency of the FVLRM is larger when the mass block is closer to the center of the FVLRM, which is similar to the simulations. When the mass block is located at position 1, the FVLRM has a maximum linear displacement velocity of 3.52 mm/s.

### 4.5. Comparison with Other Piezoelectric Actuators

Comparisons of the actuation method with other piezoelectric actuators are shown in Table 1. Several key performances of the actuators, such as their resonant operating frequency, maximum speed, working stroke, and the variable natural frequency, are mainly compared to show the improvement of the actuation method. From the table, it can be seen that the proposed actuator has advantages as follows. (a) The natural frequency of the FVLRM is lower, which can avoid serious friction problems under high-frequency signal driving, improve service life, and reduce noise. (b) The FVLRM can provide the appropriate and variable natural frequency, in order to adjust the natural frequency of the piezoelectric actuator system to adapt to the driving electrical signal. (c) The proposed FVLRM achieves two degrees of freedom movement and takes a higher speed and larger working stroke, which expands its application field.

## 5. Conclusions

An FVLRM with SRHFVS-FVPOs was proposed and systematically studied for rotor-blade-based two-degree-of-freedom actuation, which was installed on an isolation workbench. Linear motion was achieved along the *x*-axis in the positive and negative directions, while rotary motion was realized around the *z*-axis. A simulation analysis and sequence analysis of the FVLRM and SRHFVS-FVPO were performed to obtain the frequency characteristics and driving principle under different mass configurations. Moreover, an FVPO frequency regulation test was carried out and the linear and angular displacement/velocity characteristics of the FVLRM were experimentally explored in depth and in detail. The experimental results showed that the frequency of the linear and rotary motion control signals of the actuator was in the low-frequency range of 1 Hz~60 Hz. The natural frequency of the proposed actuator was adjustable, and the second-order natural frequency adjustment range was 39 Hz~54 Hz. The actuator contributed a large speed at the resonance frequency, with a working stroke of 140 mm linear movement and 360° rotation. It achieved a maximum average angular velocity of 286.9 mrad/s and a maximum linear displacement velocity of 3.52 mm/s at the working frequency of 42 Hz and voltage of 88 V, which indicates its widely applicable potential in industrial applications.

## Figures and Tables

**Table 1:** Comparison with other piezoelectric actuators.

Actuator	DOF	Resonant Operating Frequency	Maximum Speed	Working Stroke	Variable Natural Frequency
[36]	2 L + 1 R	93.5 Hz	76.7 μm/s 161.7 μrad/s	N/A	No
[38]	1 L + 1 R	662.7 Hz	N/A	95 μm 22.9 mrad	No
[40]	2 R	100 Hz	N/A	2.12 mrad 2.01 mrad	No
[41]	1 L + 1 R	1730.2 Hz	382 μm/s 200.9 mrad/s	N/A	No
This work	1 L + 1 R	42 Hz	3.52 mm/s 286.9 mrad/s	144 mm 360°	Yes

## Data Availability

Not applicable.
